# Supplementary material for: The chromatin factors SET-26 and HCF-1 oppose the histone deacetylase HDA-1 in longevity and gene regulation in C. elegans
Source: Nat Commun. 2024 Mar 14;15:2320. doi: 10.1038/s41467-024-46510-6 (PMC10940595; doi:10.1038/s41467-024-46510-6)
Supplement: Supplementary file 1 — Supplementary Information [file 41467_2024_46510_MOESM1_ESM.pdf]

Supplementary Information for Emerson et al.

The chromatin factors SET-26 and HCF-1 oppose the histone deacetylase HDA-1 in longevity and gene regulation in *C. elegans*

Supplementary Information

	Pages
Supplementary Figures	2-14
Supplementary Methods	14-18
Supplementary References	19-20

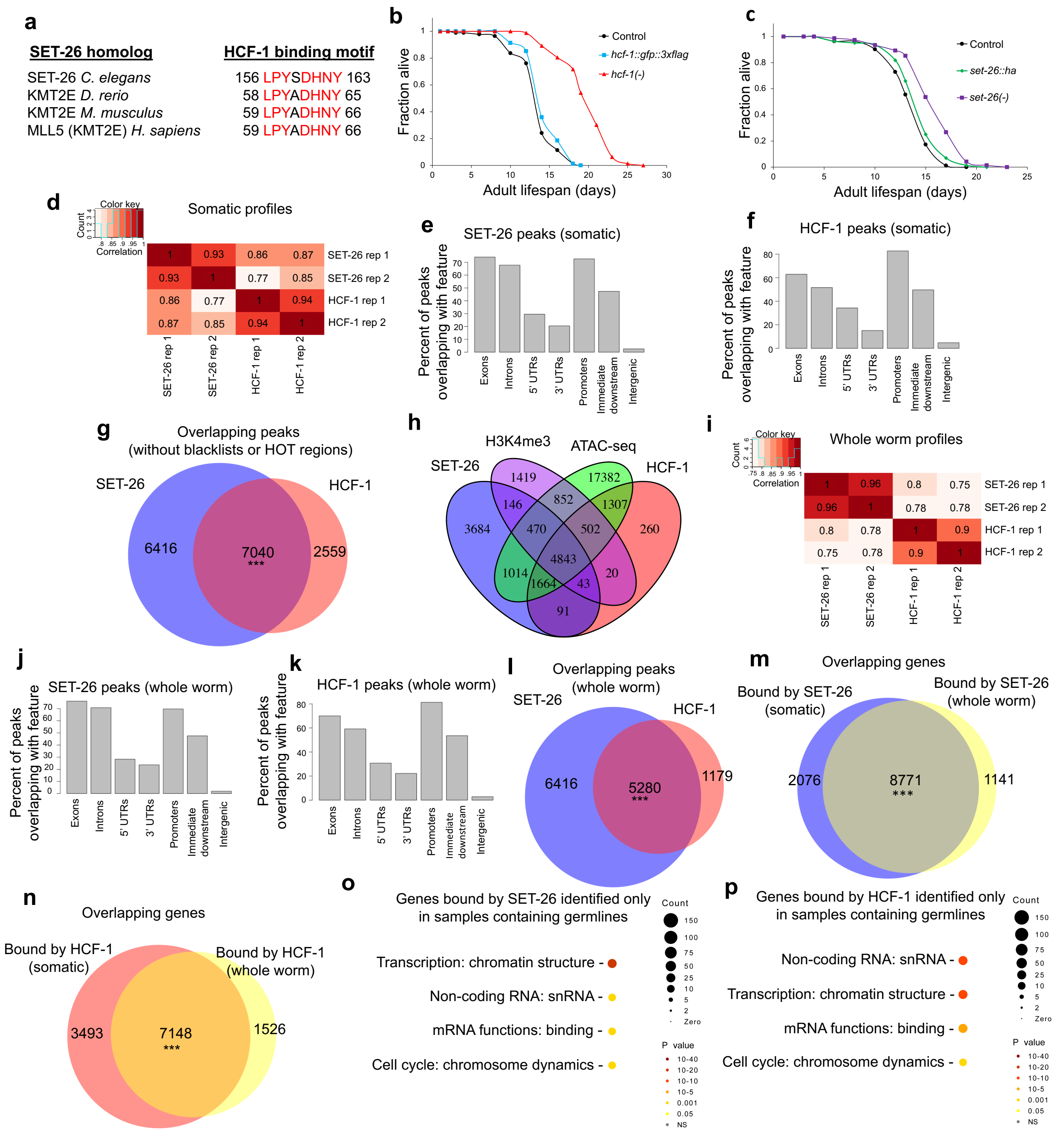

Supplementary Fig. 1: Quality control and whole worm profiles for SET-26 and HCF-1 binding. a Alignment of the HCF-1 binding motif (DHNY) and immediate upstream sequence identified in MLL5 (by Zhou et al.[1]) and the corresponding amino acid sequences showing conservation. **b-c** Survival curves for worms carrying epitope-tagged (**b**) *hcf-1* (*hcf-1::gfp::3xflag*, n=90 worms) or (**c**) *set-26* (*set-26::ha*, n=89 worms*)* compared to wildtype controls (n= 89 and 87 worms, respectively) and loss of function mutants of *hcf-1* (n= 89 worms) or *set-26* (n=89 worms) from one representative experiment. N=2. **d** Pearson's correlation of CUT&RUN profiles for SET-26 or HCF-1 binding in the *glp-1(-)* mutant (N=2). **e-f** Annotation of the proportion of somatic (**e**) SET-26 or (**f**) HCF-1 peaks that overlap the indicated features. **g** Venn diagram showing somatic SET-26 and HCF-1 peaks in the *glp-1(-)* mutant when

blacklisted regions and HOT regions are removed prior to peak calling, and peaks overlapping by 1bp or more. **h** Venn diagram showing somatic SET-26 and HCF-1 peaks in the *glp-1(-)* mutant, and their overlap by 1bp or more with H3K4me3 obtained from Pu et al.[2], and accessible ATAC-seq peaks from Jänes et al.[3]. All pairwise overlaps reached statistical significance. **I** Pearson's correlation of CUT&RUN profiles for SET-26 or HCF-1 binding in the wildtype background containing germlines (N=2). **j-k** Annotation of the proportion of whole worm (**j**) SET-26 or (**k**) HCF-1 peaks that overlap the indicated features. **l** Venn diagram showing SET-26 and HCF-1 peaks in the wildtype background, and peaks overlapping by 1bp or more. **m-n** Venn diagrams showing genes associated with (**m**) SET-26 or (**n**) HCF-1 peaks in somatic (*glp-1(-)* mutant) samples compared to whole worm (wildtype) samples. **o-p** GO term analysis (Wormcat) for the (**o**) 1,141 genes bound by SET-26 or (**p**) 1,526 genes bound by HCF-1 that were identified only in wildtype samples containing germlines and not in *glp-1(-)* mutants. *** indicates $p<1x10^{-15}$, as calculated by hypergeometric test for peak overlap (in **g, h**, and **l**) and Fisher's Test for gene overlap (in **m** and **n**). In (**o-p**), Wormcat *p* values are determined by Fisher test with FDR correction. Quantitative data are provided in Source Data; peaks and genes are provided in Supplementary Data 2.

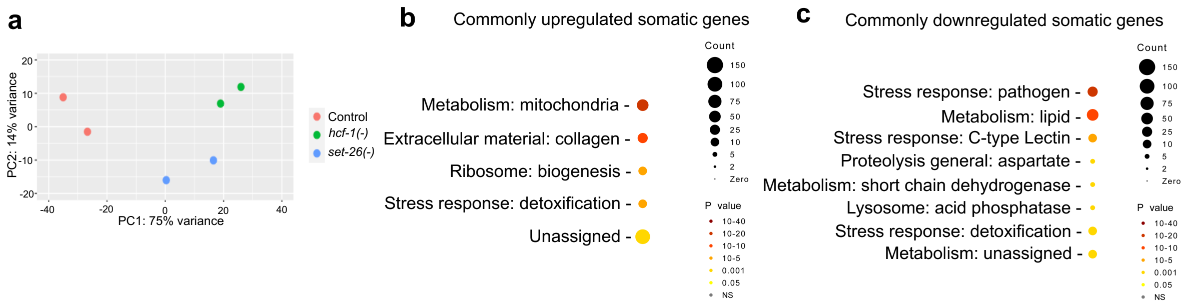

Supplementary Fig. 2: Quality control for RNA-seq analysis. a PCA plot showing correlation between RNA-seq replicates (N=2) for *glp-1(-), glp-1(-);hcf-1(-),* and *glp-1(-);set-26(-)* mutants at day 1 of adulthood. **b-c** Wormcat GO enrichment analysis for genes commonly (**b**) upregulated (485 genes) or (**c**) downregulated (602 genes) in RNA expression in both *glp-1(-);hcf-1(-),* and *glp-1(-);set-26(-)* mutants compared to single *glp-1(-)* mutants as determined in Fig. 2a and b. Wormcat *p* values are determined by Fisher test with FDR correction. Gene sets are provided in Supplementary Data 3. PC, principal component.

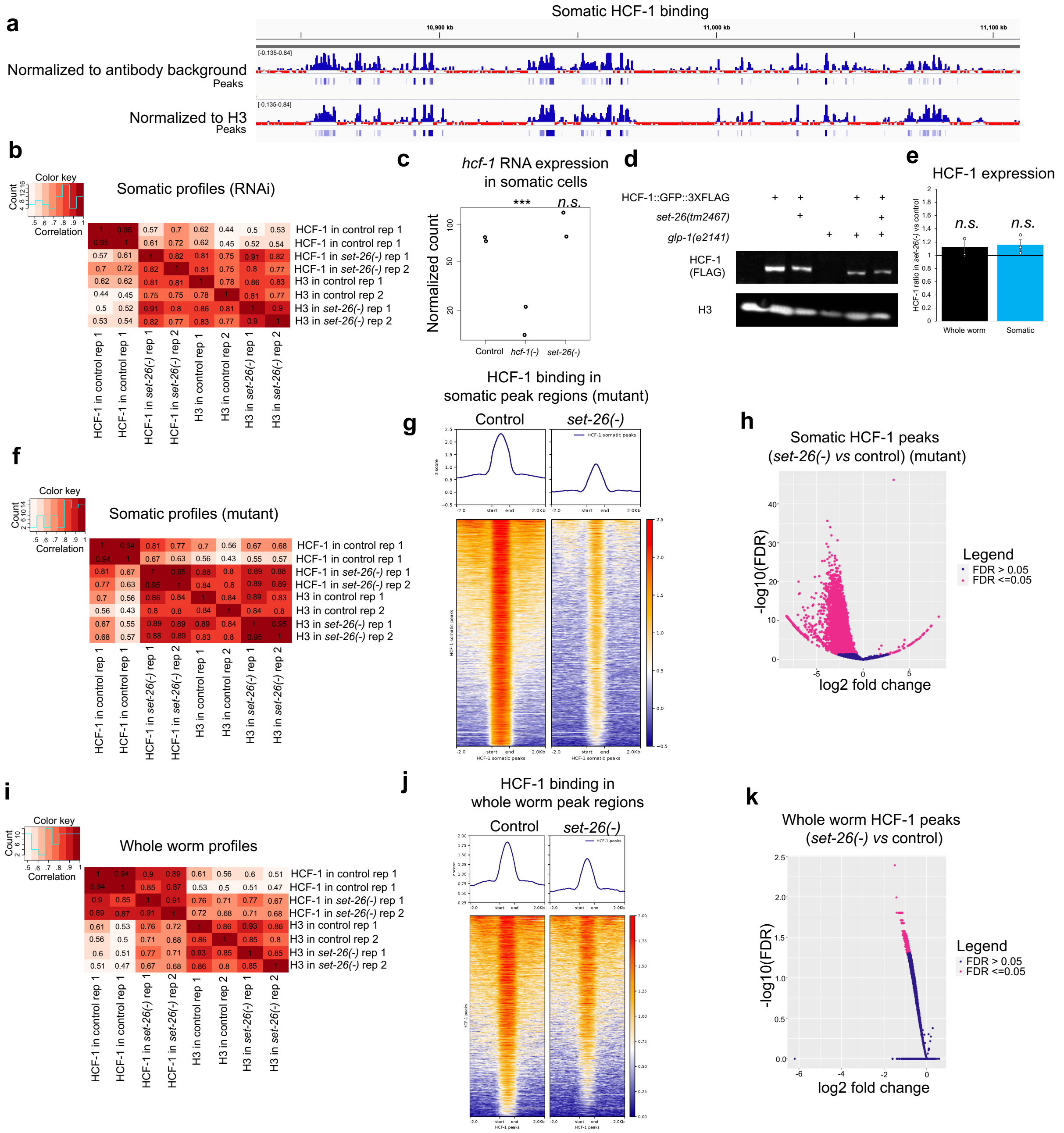

Supplementary Fig. 3: HCF-1 requires SET-26 primarily in somatic cells for its recruitment to chromatin. a IGV screenshot showing somatic HCF-1 binding (ChrV), with binding and peak calls either normalized to antibody background (top) as in Fig. 1d-e, or to H3 (bottom) as in Fig. 3a. CUT&RUN profiles (N=2) were captured using *hcf-1::gfp::3xflag* worms in either the *glp-1(-)* mutant background (top) or with *glp-1* RNAi (bottom). **b** Pearson's correlation of CUT&RUN profiles for HCF-1 or H3 in controls (*hcf-1::gfp::3xflag* worms) or *set-26(-)* mutants on *glp-1* RNAi. **c** Normalized counts of *hcf-1* expression from RNA-seq of day 1 adult *glp-1*, *glp-1(-);set-26(-)*, or *glp-1(-);hcf-1(-)* mutants. Each dot represents one replicate. **d** Representative replicate of immunoblotting experiments targeting HCF-1 or H3 in day 1 adult controls (*hcf-1::gfp::3xflag* worms) or *set-26(-)* mutants in wildtype or *glp-1(-)* mutant backgrounds. N2 and *glp-1(-)* untagged worms were used as negative controls for the FLAG antibody (lanes 1 and 4).

e Quantification of immunoblotting comparing HCF-1 levels normalized to H3 in *set-26(-)* mutants versus controls in wildtype (whole worm) or germline-less *glp-1(-)* mutant (somatic) backgrounds. N = 2-3 experiments; each dot represents one replicate. **f** Pearson's correlation of CUT&RUN profiles for HCF-1 or H3 in *glp-1(-)* single mutants or *glp-1(-);set-26(-)* double mutants (N=2). **g** Metaplot (top) and heatmap (bottom) of HCF-1 signal in somatic HCF-1 binding sites and surrounding 2kb up-and downstream in either *glp-1(-)* or *glp-1(-);set-26(-)* mutants. **h** Volcano plot of HCF-1 binding regions in *glp-1(-);set-26(-)* mutants vs. *glp-1(-)*. **i** Pearson's correlation of CUT&RUN profiles for HCF-1 or H3 control in wildtype worms or *set-26(-)* mutants grown on empty vector (E.V) RNAi (N=2). **j** Metaplot (top) and heatmap (bottom) of HCF-1 signal in whole worm HCF-1 binding sites and surrounding 2kb up-and downstream in either WT or *set-26(-)* mutants grown on E.V. **k** Volcano plot of HCF-1 binding regions in *set-26(-)* mutants vs. wildtype on E.V. In (**c**), *** indicates FDR <0.0005 and *n.s.* FDR >0.05 as determined by DESeq2. In (**e**), *n.s.* represents p>0.05 determined by two-tailed t-test; error bars represent standard errors. In (**h**) and (**k**), pink indicates FDR <=0.05 and blue indicates FDR >0.05, determined by DiffBind. Quantitative data in Source Data. Genes and peaks in Supplementary Data 4.

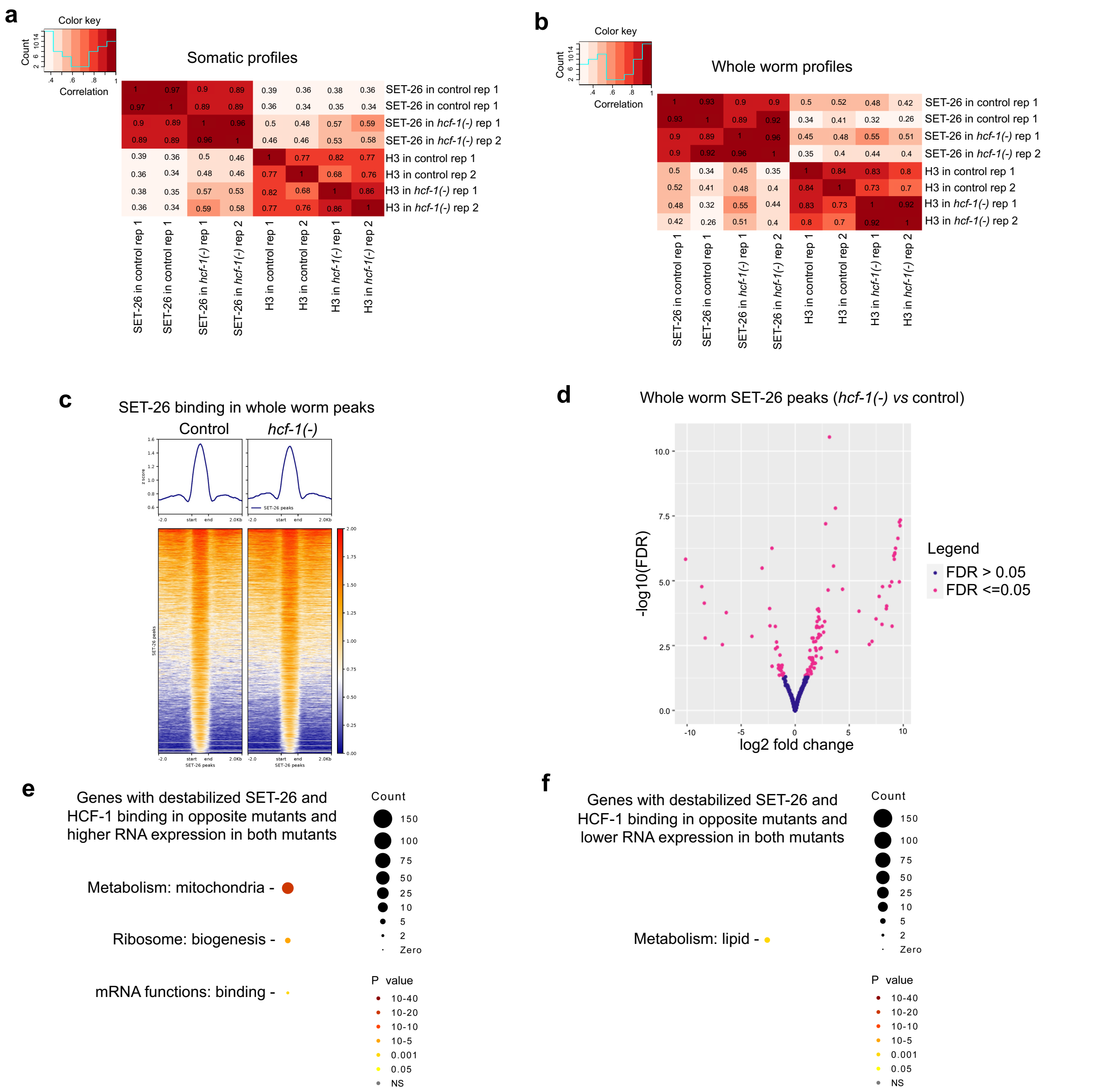

Supplementary Fig. 4: The requirement for HCF-1 in a subset of SET-26 binding sites primarily occurs in somatic cells. a-b Pearson's correlation of CUT&RUN profiles for SET-26 or H3 in controls (*set-26::ha* worms) or *hcf-1(-)* mutants grown on (**a**) *glp-1* RNAi or (**b**) E.V. control RNAi in two independent replicates. **c** Metaplot (top) and heatmap (bottom) of SET-26 signal in whole worm SET-26 binding sites and surrounding 2kb up-and downstream in either controls or *hcf-1(-)* mutants grown on E.V. control RNAi. **d** Volcano plot of SET-26 binding regions determined by DiffBind to be significantly different (pink, FDR <= 0.05) or unchanged (blue, FDR >0.05) in *hcf-1(-)* mutants compared to controls grown on E.V. control RNAi. DiffBind FDR values are calculated using DESeq2. **e-f** Wormcat GO enrichment analysis for the (**e**) 122 genes identified in Fig. 4h or (**f**) 79 genes identified in Fig. 4i that are upregulated or downregulated respectively in germline-less *set-26(-)* and *hcf-1(-)* mutants, and which display decreased somatic SET-26 and HCF-1 binding in the opposite mutant. Wormcat *p* values are determined by Fisher test with FDR correction. Gene sets and differential peaks are provided in Supplementary Data 3 and 4.

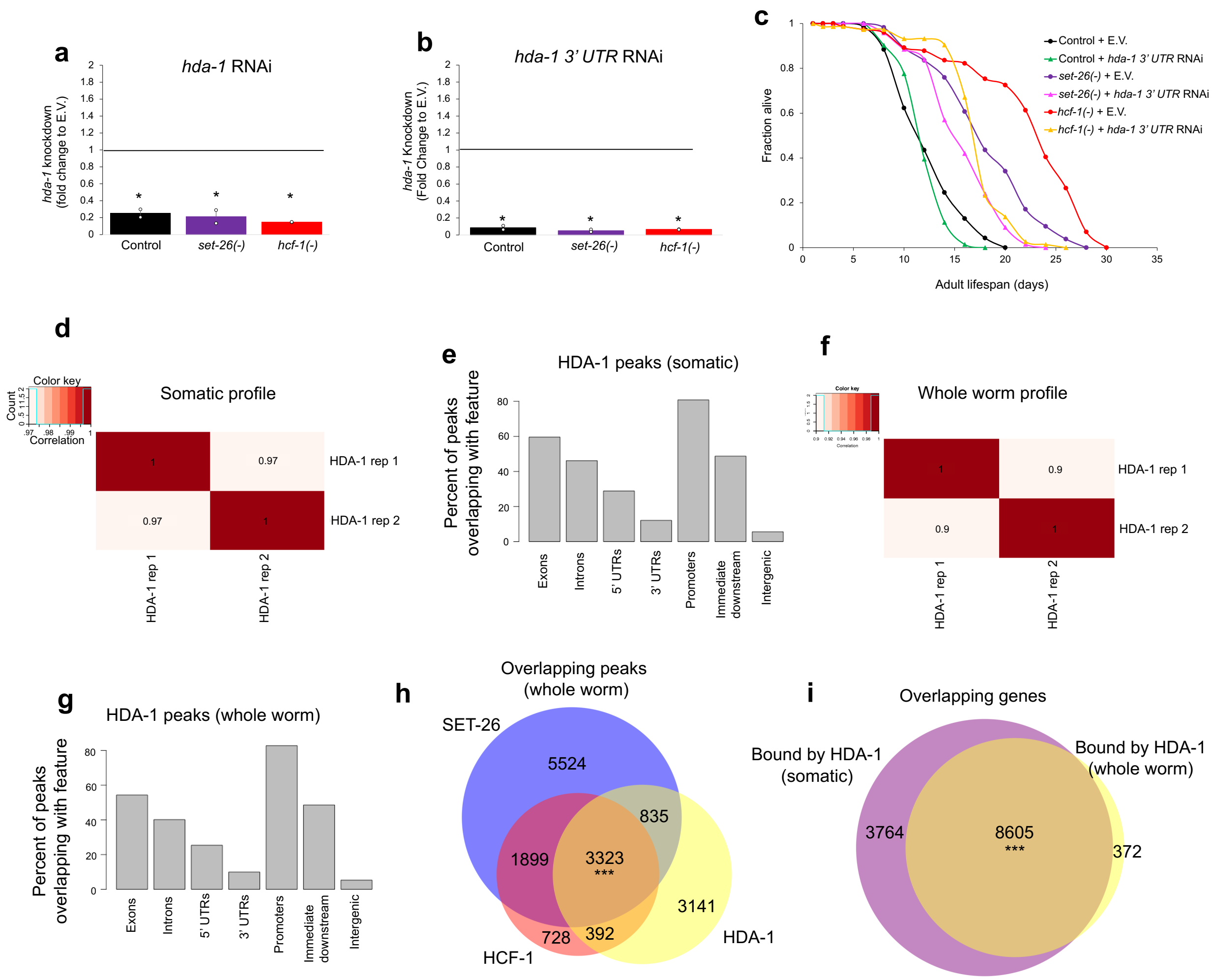

Supplementary Fig. 5: Validation of *hda-1* requirement for *set-26(-)* and *hcf-1(-)* mutant longevity and HDA-1 binding profiles. a-b *hda-1* RNA expression measured by qRT-PCR in day 3 adults treated with (**a**) *hda-1* RNAi from the Ahringer library or (**b**) newly constructed *hda-1 3' UTR* RNAi initiated on day 1 of adulthood in WT control worms, *set-26(-)* mutants, or *hcf-1(-)* mutants. Fold change represents the change in *hda-1* RNA levels in worms on *hda-1* RNAi relative to each genotype grown on E.V. RNAi. N=2 independent experiments, dots represent individual biological replicates. **c** Survival curves for wildtype controls, *set-26(-)*, and *hcf-1(-)* mutants on E.V. control RNAi and *hda-1 3' UTR* RNAi from one representative experiment, n=73, 75, 75, 74, 75, and 74 worms, respectively. *hda-1* RNAi was initiated on day 1 of adulthood. N=2 biological replicates. **d** Pearson's correlation of the CUT&RUN profiles for HDA-1 (*hda-1::gfp::ha* worms) in a *glp-1(-)* mutant background in two independent replicates. **e** Annotation of the proportion of somatic HDA-1 peak regions that overlap with the indicated genomic features. A single peak may be annotated to multiple features if it spans more than one feature. **f** Pearson's correlation of the whole-worm CUT&RUN profiles for HDA-1 (*hda-1::gfp::ha* worms) in a wildtype background in two independent replicates. **g** Annotation of the proportion of whole worm HDA-1 peak regions that overlap with the indicated genomic features. A single peak may be annotated to multiple features if it spans more than one feature. **h** Venn diagram showing SET-26, HCF-1, and HDA-1 peaks found from whole worm wildtype samples and the

3,323 peaks that overlap by 1bp or more. **i** Venn diagram showing the number of genes identified as bound by HDA-1 in somatic (*glp-1(-)* mutant) samples compared to whole worm (wildtype) samples. In (**a** and **b**) * indicates $p<0.05$ in one-tailed t-test. Error bars represent standard errors. In (**h** and **i**), *** indicates $p<1x10^{-15}$ and the overlap is higher than expected by chance, as calculated by hypergeometric test for peak overlap (in **h**) and Fisher's Test for gene overlap (in **i**). Quantitative data are provided in Source Data and gene sets are provided in Supplementary Data 2.

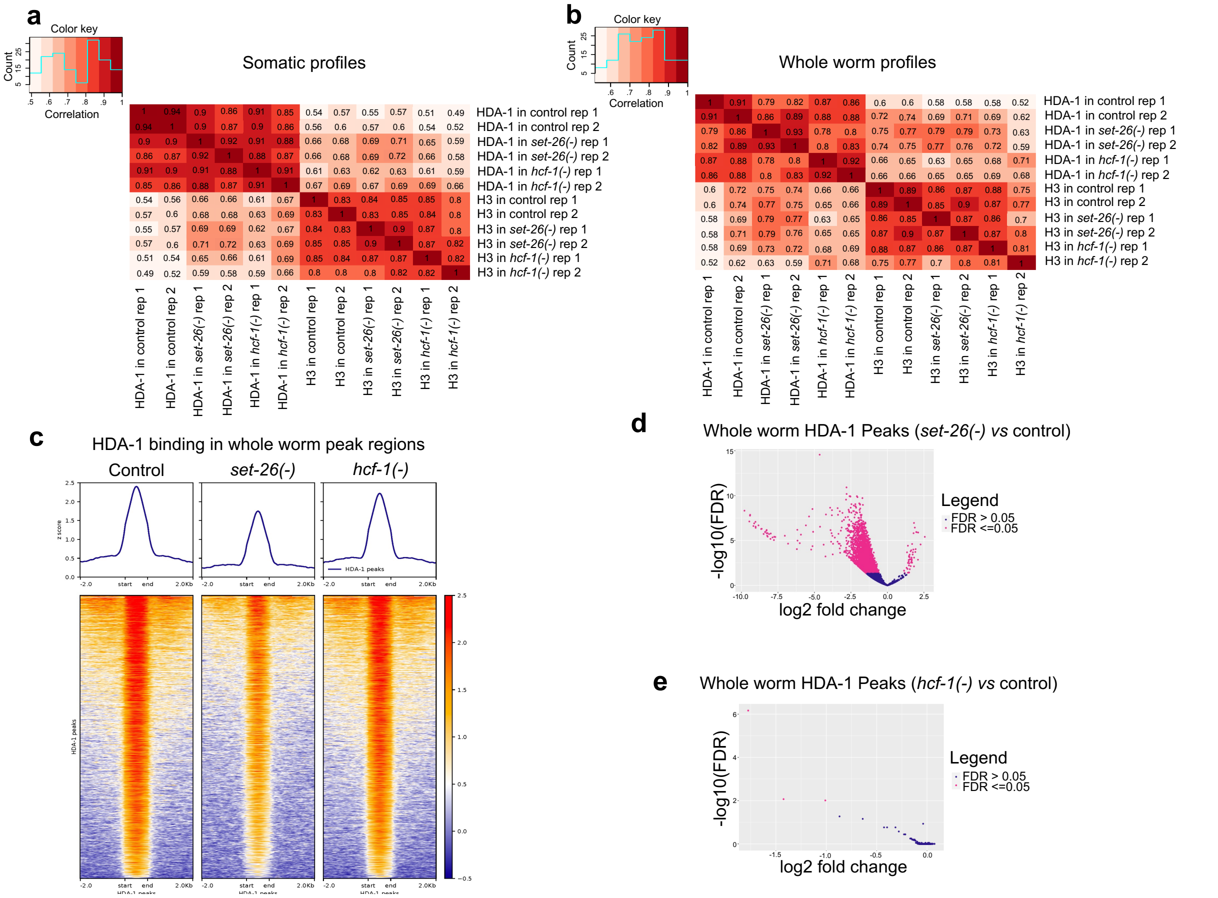

Supplementary Fig. 6: HDA-1 recruitment to chromatin is more dramatically affected by loss of *set-26* in whole worms than in germline-less worms. a-b Pearson's correlation of the CUT&RUN profiles for either HDA-1 or H3 in controls (*hda-1::gfp::ha* worms), *set-26(-)* mutants, or *hcf-1(-)* mutants grown on (**a**) *glp-1* RNAi or (**b**) E.V. control RNAi in two independent replicates. **c** Metaplot (top) and heatmap (bottom) of z-scores representing normalized HDA-1 signal in whole worm HDA-1 binding sites and surrounding 2kb up-and downstream in controls, *set-26(-)* mutants, or *hcf-1(-)* mutants grown on E.V. control RNAi. **d-e** Volcano plots of HDA-1 binding regions determined by DiffBind to be significantly different (pink, FDR <= 0.05) or unchanged (blue, FDR >0.05) in (**d**) *set-26(-)* or (**e**) *hcf-1(-)* mutants compared to controls grown on E.V. control RNAi. Diffbind FDR values in are calculated using DESeq2. Gene sets and differential peaks are provided in Supplementary Data 4.

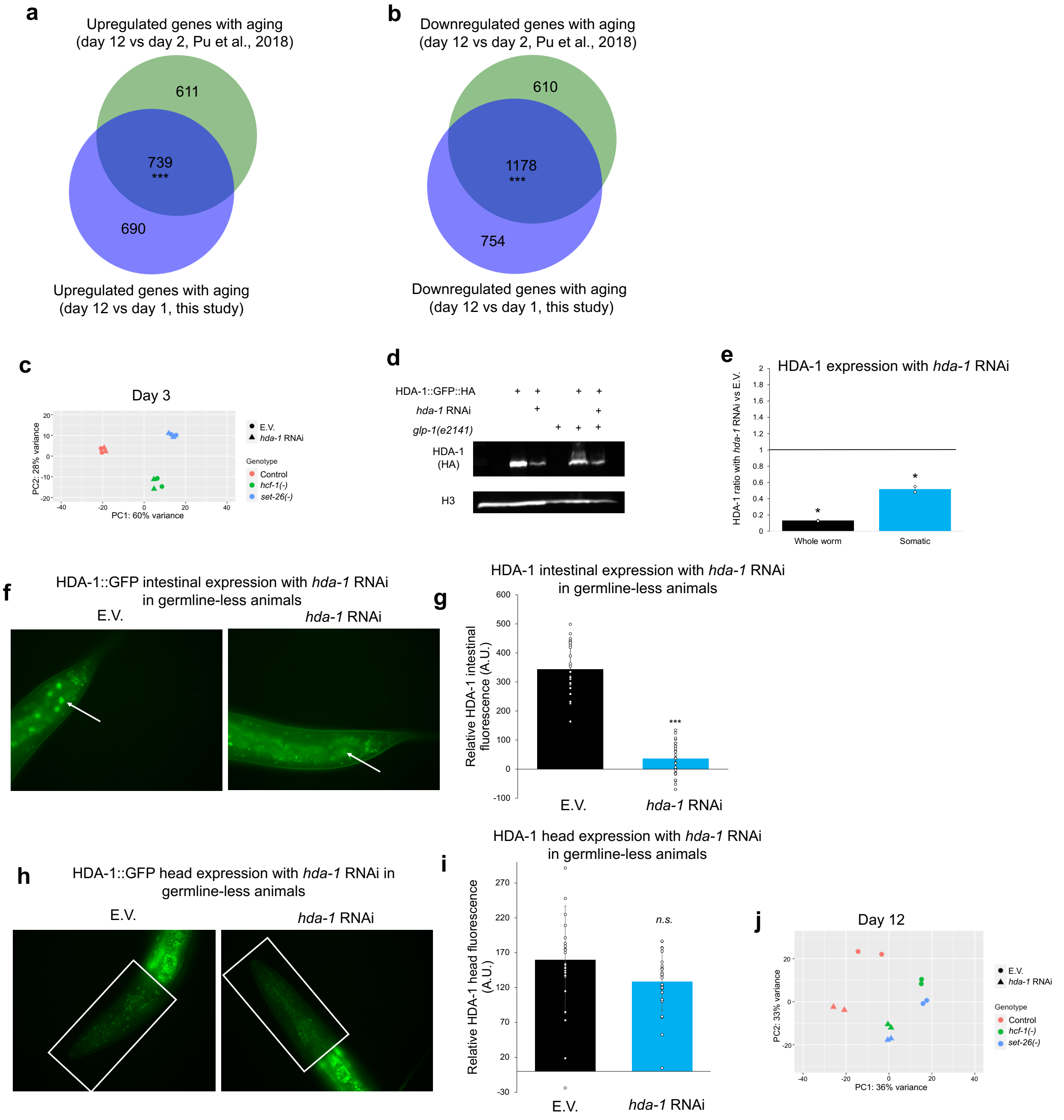

Supplementary Fig. 7. Age-related HDA-1-dependent gene expression. a-b Venn diagram showing genes (**a**) upregulated or (**b**) downregulated in RNA expression during aging in the current study or in Pu et al.[2], and the (**a**) 739 or (**b**) 1,178 common genes. **c** PCA plot showing RNA-seq replicates (N=2) for day 3 adult *glp-1(-), glp-1(-);hcf-1(-),* and *glp-1(-);set-26(-)* mutants aged on E.V. or *hda-1* RNAi initiated on day 1 of adulthood. **d** Representative replicate of immunoblotting experiments targeting HDA-1 or H3 in day 3 adults in controls (*hda-1::gfp::ha* worms) or germline-less *glp-1(-)* mutants exposed to control E.V. or *hda-1* RNAi initiated on day 1 of adulthood. N2 and *glp-1(-)* untagged worms were used as negative controls for the HA antibody (lanes 1 and 4). **e** Quantification of immunoblotting comparing HDA-1 level normalized to H3 in worms exposed to *hda-1* RNAi versus E.V. in wildtype (whole worm) or germline-less

glp-1(-) mutants (somatic). N = 2, with each dot representing an individual replicate. **f-i** Representative images and quantification of (**f**) posterior intestinal cells (n=25 and 26) or (**h**) heads (n=27 and 26) of *glp-1(-)* germline-less worms expressing the HDA-1::GFP::HA-tag as day 3 adults after being subjected to E.V. or *hda-1* RNAi since day 1 of adulthood. In **f**, arrows point to examples of intestinal cells. In (**h**), boxes outline heads. In (**g**) and (**i**), values represent the relative fluorescence of the region of interest (intestinal cell or head) compared to a nearby region without signal in the same worm. N=2, and each dot represents a measurement from an individual worm. **j** PCA plot showing RNA-seq replicates (N=2) for day 12 adult *glp-1(-), glp-1(-);hcf-1(-),* and *glp-1(-);set-26(-)* mutants aged on E.V. or *hda-1* RNAi initiated on day 1 of adulthood. In (**a**) and (**b**), *** indicates $p<1x10^{-15}$ as calculated by Fisher's Test. In (**e**), * represents $p<0.05$ as determined by one-tailed t-test and error bars represent standard errors. In (**g**) and (**i**), *** indicates $p<1x10^{-15}$ and *n.s.* indicates $p>0.05$ as calculated by two-tailed t-test and error bars represent standard deviation. Quantitative data are available in Source Data and genes are provided in Supplementary Data 3. A.U., arbitrary units. PC, principal component.

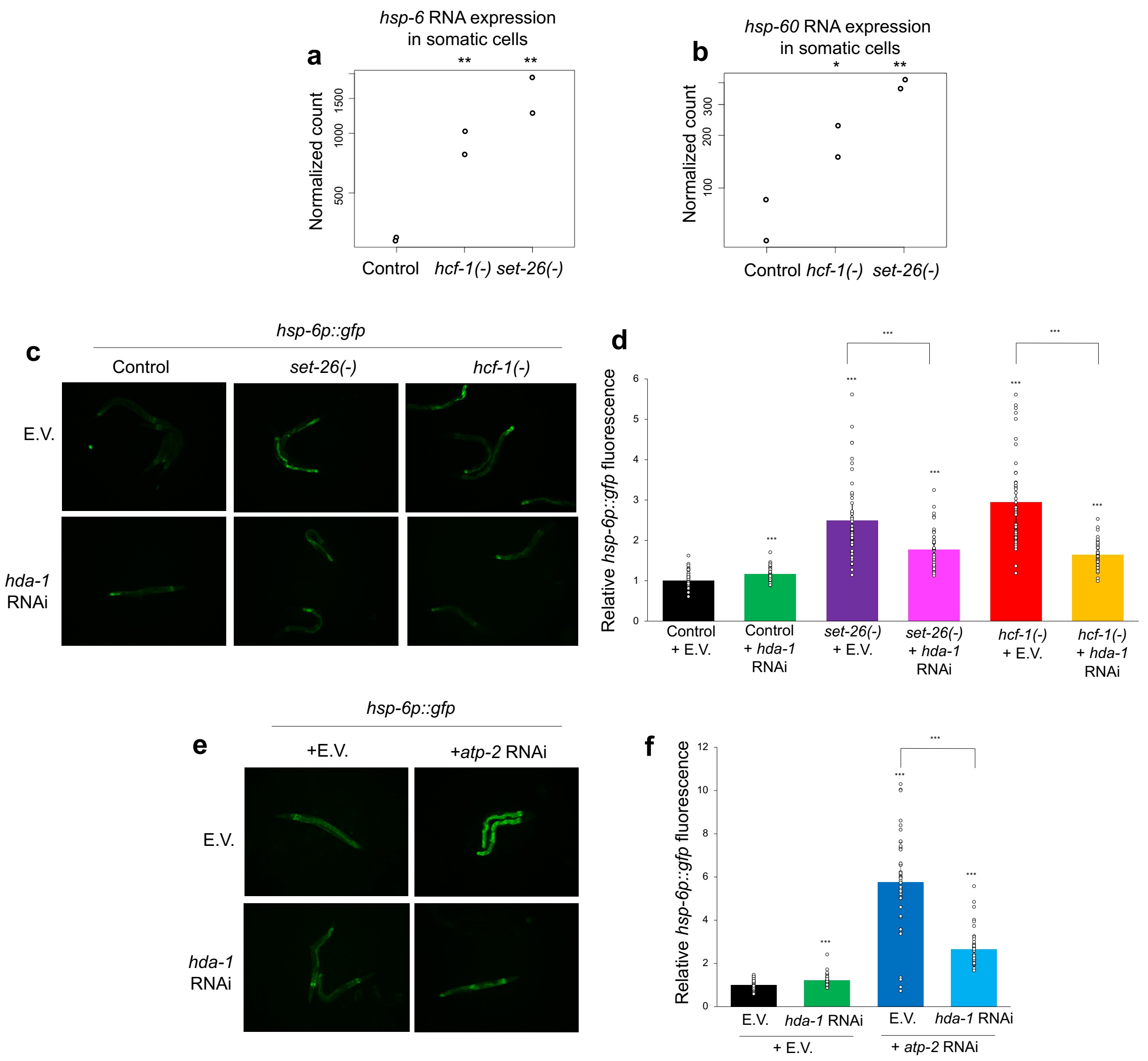

Supplementary Fig. 8. MitoUPR activation in *set-26(-)* and *hcf-1(-)* mutants. a-b Normalized read counts as determined by DESeq2 of (**a**) *hsp-6* or (**b**) *hsp-60* RNA expression from RNA-seq of day 1 adult *glp-1(-)*, *glp-1(-);set-26(-)*, and *glp-1(-);hcf-1(-)* mutants. Each dot represents one biological replicate. *hsp-6* RNA displays a fold change increase of 5.46 in *set-26(-)* mutants and 3.12 in *hcf-1(-)* mutants respectively, while *hsp-60* RNA displays a fold change increase of 5.82 in *set-26(-)* mutants and 2.79 in *hcf-1(-)* mutants respectively. **c-d** Representative images and quantification of the GFP fluorescence of young adult control worms, *set-26(-)* mutants, and *hcf-1(-)* mutants expressing *hsp-6p::gfp* grown on empty vector (E.V.) or *hda-1* RNAi from egglay (n=49, 49, 44, 43, 40, and 45 worms, respectively). **e-f** Representative images and quantification of young adult control worms expressing *hsp-6p::gfp* and grown on either E.V. or *hda-1* RNAi from egglay, having been supplemented with either E.V. control or *atp-2* RNAi bacteria to induce the mitoUPR 24h after egglay as in Shao et al.[4] (n=37, 41, 44, and 46 worms, respectively). In (**d**) and (**f**), values represent the relative fluorescence of the GFP channel compared to the channel background and autofluorescence for each worm. N=2 biological

replicates, and each dot represents a measurement from an individual worm. In (**a** and **b**), * represents *FDR<0.05* and ** represents $FDR<1x10^{-5}$ as determined by DESeq2. In (**d** and **f**), *** represents *p<0.00833*, after Bonferroni post-hoc testing following ANOVA. Quantitative data are provided in Source Data.

Supplementary Methods

CUT&RUN data analysis (continued)
In the linux environment, adaptor sequences were trimmed and low-quality reads were filtered out from sequencing files using Trim Galore! (v0.6.5), which utilizes Cutadapt (v3.4)[5] and FastQC (v0.11.8), with the settings –paired –q 20 –fastqc. Trimmed paired-end sequencing files were then aligned to the ce11/WBcel235 *C. elegans* reference genome using bowtie2 (v2.4.3)[6] with the options –very-sensitive-local –no-unal –no-mixed –no-discordant –phred33 -p 4 -I 10 -X 700. The resulting sam files were then converted to bam files using 'samtools view' command in SAMTools (v1.14)[7] with the options -hSb -F 4. Since keeping duplicated reads is recommended for CUT&RUN, they were not removed[8,9]. The individual bam files corresponding to two biological replicates were considered both individually (for correlation analysis and DiffBind) and combined (for heatmaps, metaplots, and visualization on IGV (v2.4.19)[10]) using the 'samtools merge' command in SAMTools for downstream analysis. For Supplementary Fig. 1g, ce11 blacklisted regions were obtained from the ENCODE lists[11] and removed using the bedtools intersect -v command in BEDtools(v2.29.2)[12]. Similarly, for Supplementary Fig. 1g, HOT regions were obtained from Chen et al.[13], converted to ce11 coordinates using the Lift Genome Annotations tool in the UCSC Genome Browser[14], and removed using the bedtools intersect -v command. Bam files were then sorted using 'samtools sort' command and indexed using the 'samtools index' command in SAMTools using default settings.

Indexed bam files were used for narrow peak calling with MACS2 (v2.1.4)[15] using the settings -f BAM - g ce –call-summits –keep-dup all -q 0.01 -m 5 50 –nomodel. When calling peaks for factor binding to characterize binding sites (as in Fig. 1), peaks of each factor were called using combined replicates of factor binding against a control bam file of two combined biological replicates of experiments targeting the tag of interest in the N2 background (i.e. CUT&RUN with an anti-FLAG or anti-HA antibody in N2 or *glp-1(-)*, which produces background antibody binding tracks). When calling peaks for factor binding to directly compare the degree of binding in two genotypes (as in Fig. 3, 4, and 6), peaks were called with a control file representing H3 binding in each genotype (i.e. *hcf-1::gfp::3xflag set-26(-)* strain subjected to H3 CUT&RUN in parallel to FLAG). This process was repeated with the individual bam files for each replicate and the merged bam files representing both replicates as discussed above. The number of peaks discussed represents the number of unique peaks called (after removing duplicate locations with multiple summits called by MACS2) and results from peak calling using merged bam files.

To generate bigwig files for visualization, combined bam files from both biological replicates were put through the 'bamCompare' command in deepTools (v3.3)[16] with the settings –binSize 20 –operation log2 –scaleFactors Method None –normalizeUsing CPM –numberOfProcessors 8 –outFileFormat bigwig –smoothLength 60 –extendReads –centerReads, and with –bamfile2

representing a bam file with combined replicates of CUT&RUN experiments targeting either the tagged factor of interest in untagged control worms (N2 or *glp-1(-)*) or H3, as indicated in figure legends.

To determine the correlation between biological replicates, the mapped read count in 2 kb sliding windows was obtained for each bam file corresponding to a single replicate using the command 'bedtools multicov' in BEDtools (v2.29.2)[12] using a reference of the ce11/WBcel235 genome broken into 2 kb windows generated with the 'bedtools makewindows' command. The readcount of each sequencing file in each 2 kb window was uploaded into RStudio, converted into log10, and then the Pearson's correlation was calculated between each replicate using the 'cor' function in R. The correlations were saved in a matrix and plotted using the 'heatmap.2' function within the gplots package (v3.1.3) in R.

To find overlapping peaks, peak regions called by MACS2 for factor binding vs antibody background were uploaded into the R environment in the package ChIPpeakAnno (v3.28.1)[17] and used as input for the command 'findOverlapsOfPeaks' with the settings 'minoverlap=1, connectedPeaks="keepAll"). The overlapping peaks were then plotted using the 'makeVennDiagram' command, and a hypergeometric test was performed to test the significance of overlap between the peaksets using the option 'totalTest=30000'.

To annotate the genomic features associated with peaks, the same peaks were used as input for the 'assignChromosomeRegion' command in ChIPpeakAnno using the default settings and the annotation data of TxDb.Celegans.UCSC.ce11.ensGene downloaded into the R environment from Bioconductor (v3.15). Note that a single peak may be annotated to multiple features if it spanned more than one feature. To produce density plots of factor binding around the TSS, the same peak regions were used as input for the 'binOverFeature' command in ChIPpeakAnno, using the default settings with radius = 3000 and the annotation data of TxDb.Celegans.UCSC.ce11.ensGene. To produce density plots of SET-26 and HCF-1 binding within common peak regions, the overlapping peaks identified from the 'findOverlapsOfPeaks' command were recentered with a width of 1. The factor binding signal extracted from bigwig files containing combined replicates of normalized factor binding versus antibody background were used as input (cvglists) for the 'featureAlignedSignal' command in ChIPpeakAnno, with the recentered overlapping peaks as the reference frame of which to plot (feature.gr).

To annotate CUT&RUN peaks with associated genes, the peaks of each factor binding versus antibody background were uploaded to ChIPpeakAnno and used as input for the 'annotatePeakInBatch' function in ChIPpeakAnno, with the settings of 'output = "nearestBiDirectionalPromoters", bindingRegion = c(-2000, 500)' and the annotation data of TxDb.Celegans.UCSC.ce11.ensGene. The process was repeated for regions found to be significantly differentially bound by DiffBind. BioVenn (www.biovenn.nl) was used for generating Venn Diagrams[18], and the significance of overlapping genes were determined by Fisher's exact test calculated in RStudio. WormCat 2.0 (www.wormcat.com) was used for gene ontology enrichment analysis, where the p values are determined by Fisher's test with FDR correction[19].

To produce z-score normalized bigwig files of factor binding in different genotypes to use as input for heatmaps and metaplots, combined bam files from both biological replicates of factor binding were first converted to bedgraphs using the 'bamCompare' command with the settings –binSize 20 –operation log2 –scaleFactorsMethod readCount –numberOfProcessors 8 –outFileFormat bedgraph –smoothLength 60 –extendReads, and with –bamfile2 representing a bam file with combined replicates of CUT&RUN experiments targeting H3 in parallel in the same genotype. The resulting log2 signal bedgraph files were then uploaded into R (v4.1.2) using RStudio (v2022.02.0+443) and converted into z-scores using the 'scale' function in R. Z-score converted bedgraphs were uploaded back into the Linux environment, sorted using the command 'do sort -k1,1 -k2,2n', and converted to bigwig files using the UCSC bedGraphToBigWig (v4) program with default settings.

To generate heatmaps and metaplots, computeMatrix from deepTools was used to generate a standardized matrix of z-score factor binding in predetermined peak regions with the sub-command 'scale-regions' to scale all peak regions to the same size for visualization, with settings -b 2000 -a 2000 –binSize 20 –sortRegions keep –regionBodyLength 1000 -p 4 -p max/2. The bed files of predetermined peak regions (--regionsFileName argument) were generated from MACS2 peak calling of factor vs antibody background (e.g. FLAG signal in the *hcf-1::gfp::3xflag* strain vs FLAG signal in N2) in combined replicates as described above. The z-score normalized bigwig files generated above were used as the files containing scores to be plotted (--scoreFileName argument). The resulting matrix files were used as input for the 'plotHeatmap' command in deepTools.

To compare differentially bound regions between two genotypes, sorted bam files of factor binding and control immunoprecipitations (against H3) and peak calls from MACS2 of factor binding against H3 controls for each of two biological replicates were read into the R environment and analyzed using the DiffBind package (v3.4.11)[20] with the comparison: (factor binding in mutant - H3 binding in mutant) - (factor binding in control - H3 binding in control). The standard DiffBind workflow was used, with dba.count settings of 'minOverlap=2, bUseSummarizeOverlaps = TRUE', and Volcano plots and p values generated using DBA_DESEQ2 to assess significance. Regions were considered significantly differentially bound if they were assigned an FDR value of less than 0.05 by the DESeq2 algorithm utilized by DiffBind.

ChIP-seq data analysis
ChIP-seq data for H3K4me3 and H3 in day 2 adult *glp-1(e2141)* mutant worms were downloaded from GEO series GSE101964[2] [https://www.ncbi.nlm.nih.gov/geo/query/acc.cgi?acc=GSE101964]. The data were processed similarly to the CUT&RUN data above, with minor differences to account for single-end sequencing data and the ChIP-seq technique. Trim Galore! (v0.6.5), which utilizes Cutadapt (v4.1)[5] and FastQC (v0.12.1) was used in single-end mode with the settings -q 20 –fastqc. Alignment using bowtie2 (v2.4.3)[6] was used in single-end mode with default options. Multimapped reads were excluded during sam to bam conversion using the 'samtools view' command with the options -hSb -F 4 -q 2, and duplicates were removed using the 'samtools

rmdup' command in SAMTools (v1.15.1)[7]. Bam files were sorted, replicates were merged as described above, and files were indexed as described above. Narrow peaks were called for H3K4me3 against H3 control using MACS2 (v2.2.7.1)[15] with the settings described above.

ATAC-seq data analysis
ATAC-seq data for accessible regions in young adult *glp-1(e2144)* mutants were downloaded from GEO series GSE114439[3] [https://www.ncbi.nlm.nih.gov/geo/query/acc.cgi?acc=GSE114439]. In the linux environment, adaptors were trimmed and low-quality reads were removed using Trim Galore! (v0.6.5), which utilizes Cutadapt (v4.1)[5] and FastQC (v0.12.1) with the settings -nextseq 20 –fastqc. Trimmed files were then aligned to the ce11/Wbcel235 *C. elegans* reference genome using bwa (v0.7.17)[21] with the settings mem -M. Resulting sam files were converted to bam using 'samtools view' command in SAMTools (v1.15.1)[7] with the options -@ 24 -b -h -F 0x0100. Bam files were sorted and indexed as above and mitochondrial DNA was removed using the 'samtools view' command. Blacklisted regions were removed as described above using the 'bedtools intersect' command, and duplicates were marked using 'MarkDuplicates' in Picard (v2.26.1) (http://broadinstitute.github.io/picard/) and removed using the 'samtools view' command. Replicates were merged and indexed as described above, and used for narrow peak calling in MACS2 (v2.2.7.1)[15] with the options -f BAM –gsize ce -q 0.05 –call-summits.

Fluorescence imaging
2% agarose pads in M9 were prepared on glass slides for imaging. 5-10μl of 2 mM levamisole was added to the top of the agarose pad, 5-20 worms were picked into the levamisole, and a cover slip was added. Fluorescence was detected and captured using a Leica DM 5000B compound microscope and Leica LAX software.

Fluorescence quantification
Fluorescence images were quantified using Fiji (version 2.1.0/1.53c)[22] using the mean gray value measurement tool. Briefly, the area of interest was manually selected for each image. For *hsp-6p::gfp* quantification, this was the entire worm. For HDA-1::GFP knockdown imaging, this was an individual intestinal cell or head region (ending immediately before the anterior intestine). The fluorescence of this region was quantified with Fiji. Next, background measurements were taken for normalization. For *hsp-6p::gfp* quantification, the background intensity of the GFP channel was taken (a region with no worms present), as well as the intensity of the worm in an autofluorescent channel and the background of the autofluorescent channel (a region with no worms present). To analyze the data for *hsp-6p::gfp* images, the GFP values of each worm were normalized to that worm's autofluorescence, while taking into account the background of each channel. Before plotting, each worm's fluorescent value was normalized to the average of the control's fluorescent value to allow for the y axis to represent relative fluorescence to control. Final plotted relative GFP values for *hsp-6p::gfp* imaging represent the following: ([GFP of the worm – GFP channel background] / [Autofluorescence of the worm – Autofluorescence channel background]) / average normalized fluorescent value in controls). For HDA-1::GFP knockdown imaging, background levels were measured on the same image as the region of interest, using an adjacent region of the worm not expressing GFP

(either nearby the intestine or in the head). Final plotted relative GFP values for HDA-1::GFP imaging represent the following: Fluorescence of the region of interest – Fluorescence of a nearby background region. Two biological replicates were conducted for each fluorescent imaging experiment, and individual worms from both replicates were pooled together to calculate statistical significance using either an ANOVA with post-hoc Bonferroni correction (for *hsp-6p::gfp* experiments), or two-tailed t-tests (for HDA-1::GFP knock-down experiments).

Supplementary References

1. Zhou, P. *et al.* Mixed lineage leukemia 5 (MLL5) protein regulates cell cycle progression and E2F1-responsive gene expression via association with host cell factor-1 (HCF-1). *J. Biol. Chem.* **288**, 17532–17543 (2013).
2. Pu, M., Wang, M., Wang, W., Velayudhan, S. S. & Lee, S. S. Unique patterns of trimethylation of histone H3 lysine 4 are prone to changes during aging in Caenorhabditis elegans somatic cells. *PLoS Genet.* **14**, e1007466 (2018).
3. Jänes, J. *et al.* Chromatin accessibility dynamics across C. elegans development and ageing. *eLife* vol. 7 Preprint at https://doi.org/10.7554/elife.37344 (2018).
4. Shao, L.-W. *et al.* Histone deacetylase HDA-1 modulates mitochondrial stress response and longevity. *Nat. Commun.* **11**, 4639 (2020).
5. Martin, M. Cutadapt removes adapter sequences from high-throughput sequencing reads. *EMBnet.journal* **17**, 10–12 (2011).
6. Langmead, B. & Salzberg, S. L. Fast gapped-read alignment with Bowtie 2. *Nat. Methods* **9**, 357–359 (2012).
7. Li, H. *et al.* The Sequence Alignment/Map format and SAMtools. *Bioinformatics* **25**, 2078–2079 (2009).
8. Fu, Y., Wu, P.-H., Beane, T., Zamore, P. D. & Weng, Z. Elimination of PCR duplicates in RNA-seq and small RNA-seq using unique molecular identifiers. *BMC Genomics* **19**, 531 (2018).
9. Zhu, Q., Liu, N., Orkin, S. H. & Yuan, G.-C. CUT&RUNTools: a flexible pipeline for CUT&RUN processing and footprint analysis. *Genome Biol.* **20**, 192 (2019).
10. Robinson, J. T. *et al.* Integrative genomics viewer. *Nat. Biotechnol.* **29**, 24–26 (2011).
11. Amemiya, H. M., Kundaje, A. & Boyle, A. P. The ENCODE Blacklist: Identification of Problematic Regions of the Genome. *Sci. Rep.* **9**, 9354 (2019).
12. Quinlan, A. R. & Hall, I. M. BEDTools: a flexible suite of utilities for comparing genomic

features. *Bioinformatics* **26**, 841–842 (2010).

13. Chen, R. A.-J. *et al.* Extreme HOT regions are CpG-dense promoters in C. elegans and humans. *Genome Res.* **24**, 1138–1146 (2014).
14. Kent, W. J. *et al.* The human genome browser at UCSC. *Genome Res.* **12**, 996–1006 (2002).
15. Zhang, Y. *et al.* Model-based analysis of ChIP-Seq (MACS). *Genome Biol.* **9**, R137 (2008).
16. Ramírez, F., Dündar, F., Diehl, S., Grüning, B. A. & Manke, T. deepTools: a flexible platform for exploring deep-sequencing data. *Nucleic Acids Res.* **42**, W187–91 (2014).
17. Zhu, L. J. *et al.* ChIPpeakAnno: a Bioconductor package to annotate ChIP-seq and ChIP-chip data. *BMC Bioinformatics* **11**, 237 (2010).
18. Hulsen, T., de Vlieg, J. & Alkema, W. BioVenn - a web application for the comparison and visualization of biological lists using area-proportional Venn diagrams. *BMC Genomics* **9**, 488 (2008).
19. Holdorf, A. D. *et al.* WormCat: An Online Tool for Annotation and Visualization of Caenorhabditis elegans Genome-Scale Data. *Genetics* **214**, 279–294 (2020).
20. Ross-Innes, C. S. *et al.* Differential oestrogen receptor binding is associated with clinical outcome in breast cancer. *Nature* **481**, 389–393 (2012).
21. Li, H. & Durbin, R. Fast and accurate short read alignment with Burrows–Wheeler transform. *Bioinformatics* **25**, 1754–1760 (2009).
22. Schindelin, J. *et al.* Fiji: an open-source platform for biological-image analysis. *Nat. Methods* **9**, 676–682 (2012).
