# Supplementary material for: The chromatin factors SET-26 and HCF-1 oppose the histone deacetylase HDA-1 in longevity and gene regulation in C. elegans
Source: Nat Commun. 2024 Mar 14;15:2320. doi: 10.1038/s41467-024-46510-6 (PMC10940595; doi:10.1038/s41467-024-46510-6)
Supplement: Supplementary file 3 — Description of Additional Supplementary Files [file 41467_2024_46510_MOESM3_ESM.pdf]

Description of Additional Supplementary Files

File Name: Supplementary Data 1
Description: Relative abundance of putative HCF-1 interactors identified through immunoprecipitation-mass spec experiments

File Name: Supplementary Data 2
Description: CUT&RUN binding sites (peaks and genes) under basal conditions in *C. elegans*

File Name: Supplementary Data 3
Description: Integration of RNA-seq and CUT&RUN data in *C. elegans* mutants

File Name: Supplementary Data 4
Description: CUT&RUN binding sites (peaks and genes) in mutant backgrounds in *C. elegans*

File Name: Supplementary Data 5
Description: *C. elegans* strains, primer sequences, and antibodies used in this study
