# Supplementary material for: The chromatin factors SET-26 and HCF-1 oppose the histone deacetylase HDA-1 in longevity and gene regulation in C. elegans
Source: Nat Commun. 2024 Mar 14;15:2320. doi: 10.1038/s41467-024-46510-6 (PMC10940595; doi:10.1038/s41467-024-46510-6)
Supplement: Supplementary file 9 — Reporting Summary [file 41467_2024_46510_MOESM9_ESM.pdf]

nature portfolio

Corresponding author(s): Siu Sylvia Lee

Last updated by author(s): Feb 21, 2024

Reporting Summary

Nature Portfolio wishes to improve the reproducibility of the work that we publish. This form provides structure for consistency and transparency in reporting. For further information on Nature Portfolio policies, see our Editorial Policies and the Editorial Policy Checklist.

Statistics

For all statistical analyses, confirm that the following items are present in the figure legend, table legend, main text, or Methods section.

n/a	Confirmed	
☐	☒	The exact sample size (*n*) for each experimental group/condition, given as a discrete number and unit of measurement
☐	☒	A statement on whether measurements were taken from distinct samples or whether the same sample was measured repeatedly
☐	☒	The statistical test(s) used AND whether they are one- or two-sided<br>*Only common tests should be described solely by name; describe more complex techniques in the Methods section.*
☒	☐	A description of all covariates tested
☐	☒	A description of any assumptions or corrections, such as tests of normality and adjustment for multiple comparisons
☐	☒	A full description of the statistical parameters including central tendency (e.g. means) or other basic estimates (e.g. regression coefficient) AND variation (e.g. standard deviation) or associated estimates of uncertainty (e.g. confidence intervals)
☐	☒	For null hypothesis testing, the test statistic (e.g. *F*, *t*, *r*) with confidence intervals, effect sizes, degrees of freedom and *P* value noted<br>*Give P values as exact values whenever suitable.*
☒	☐	For Bayesian analysis, information on the choice of priors and Markov chain Monte Carlo settings
☒	☐	For hierarchical and complex designs, identification of the appropriate level for tests and full reporting of outcomes
☒	☐	Estimates of effect sizes (e.g. Cohen's *d*, Pearson's *r*), indicating how they were calculated

Our web collection on statistics for biologists contains articles on many of the points above.

Software and code

Policy information about availability of computer code

Data collection	C. elegans mRNA sequencing reads were obtained by preparing mRNA sequencing libraries and sequencing through Illumina NextSeq 500.<br>C. elegans DNA sequencing reads corresponding to binding regions of the chromatin factors SET-26, HCF-1, and HDA-1 were obtained by preparing CUT&RUN libraries and sequencing through Illumina NextSeq 500.
Data analysis	CUT&RUN and mRNA sequencing data were analyzed using software as described in the manuscript. No custom software was generated for this study. The versions used are described in the manuscript and are included here.<br><br>Software analysis tools for CUT&RUN analysis included:<br>Trim Galore! (v0.6.5) which utilizes Cutadapt (v3.4) and FastQC (v0.11.8)<br>bowtie2 (v2.4.3)<br>SAMTools (v1.14)<br>BEDtools(v2.29.2)<br>MACS2 (v2.1.4)<br>deepTools (v3.3)<br>UCSC bedGraphToBigWig (v4)<br>UCSC Lift Genome Annotations (https://genome.ucsc.edu/cgi-bin/hgLiftOver)<br><br>In addition, software analysis tools for RNA-seq analysis included:<br>Trim Galore! (v0.6.5) which utilizes Cutadapt (v3.4) and FastQC (v0.11.8)<br>STAR (v2.7.9a)

In addition, software analysis tools used for ChIP-seq analysis included:
Trim Galore! (v0.6.5), which utilizes Cutadapt (v4.1) and FastQC (v0.12.1)
bowtie2 (v2.4.3)
SAMTools (v1.15.1)

In addition, software analysis tools used for ATAC-seq analysis included:
Trim Galore! (v0.6.5), which utilizes Cutadapt (v4.1) and FastQC (v0.12.1)
bwa (v0.7.17)
SAMTools (v1.15.1)
Picard (v2.26.1); MACS2 (v2.2.7.1)

R PACKAGES - R (v4.1.2) using RStudio (v2022.02.0+443), used for any/all omics analysis:
gplots package (v3.1.3)
ChIPpeakAnno (v3.28.1)
Bioconductor (v3.15)
DiffBind (v3.4.11)
DESeq2 (v1.34.0)
ComplexHeatmap (v2.10.0)
ggplot2 (v3.3.6)

VISUALIZATION
IGV (v2.4.19)
BioVenn (www.biovenn.nl)
WormCat 2.0 (www.wormcat.com)

Excel (version 16.66.1) was used for all other statistics.

For manuscripts utilizing custom algorithms or software that are central to the research but not yet described in published literature, software must be made available to editors and reviewers. We strongly encourage code deposition in a community repository (e.g. GitHub). See the Nature Portfolio guidelines for submitting code & software for further information.

Data

Policy information about availability of data

All manuscripts must include a data availability statement. This statement should provide the following information, where applicable:

- Accession codes, unique identifiers, or web links for publicly available datasets
- A description of any restrictions on data availability
- For clinical datasets or third party data, please ensure that the statement adheres to our policy

CUT&RUN and RNA-seq raw sequencing data, CUT&RUN bigwig files for visualization, and the RNA-seq read count matrix have been deposited in NCBI's Gene Expression Omnibus and are accessible through GEO series accession number GSE224075 [https://www.ncbi.nlm.nih.gov/geo/query/acc.cgi?acc=GSE224075] (RNA-seq), GSE224076 [https://www.ncbi.nlm.nih.gov/geo/query/acc.cgi?acc=GSE224076] (CUT&RUN) and can be found as SuperSeries GSE224078 [https://www.ncbi.nlm.nih.gov/geo/query/acc.cgi?acc=GSE224078]. IP-Mass spec proteomics data from the two independent experiments have been deposited to the ProteomeXchange Consortium via the PRIDE partner repository with the dataset identifier PXD047509 [https://proteomecentral.proteomexchange.org/cgi/GetDataset?ID=PXD047509] (performed by co-author C.G.R.) and PXD047247 [https://proteomecentral.proteomexchange.org/cgi/GetDataset?ID=PXD047247] (performed by co-author M.Z.). ChIP-seq data of H3K4me3 and H3 in day 2 adult glp-1(e2141) mutants were downloaded from GEO series GSE101964 [https://www.ncbi.nlm.nih.gov/geo/query/acc.cgi?acc=GSE101964] 35. ATAC-seq data of accessible regions in young adult glp-1(e2144) mutants were downloaded from GEO series GSE114439 [https://www.ncbi.nlm.nih.gov/geo/query/acc.cgi?acc=GSE114439] 36. Differentially expressed genes during aging were downloaded directly from Pu. et al.35. Blacklisted regions were obtained from ENCODE lists32 and HOT regions were obtained from Chen et al.33. For genome alignment, the ce11/WBcel235 C. elegans reference genome was downloaded from Ensembl (https://useast.ensembl.org/Caenorhabditis_elegans/Info/Index).

Research involving human participants, their data, or biological material

Policy information about studies with human participants or human data. See also policy information about sex, gender (identity/presentation), and sexual orientation and race, ethnicity and racism.

Reporting on sex and gender	Human participants not used.
Reporting on race, ethnicity, or other socially relevant groupings	Human participants not used.
Population characteristics	Human participants not used.
Recruitment	Human participants not used.
Ethics oversight	Human participants not used.

Note that full information on the approval of the study protocol must also be provided in the manuscript.

Field-specific reporting

Please select the one below that is the best fit for your research. If you are not sure, read the appropriate sections before making your selection.

☒ Life sciences ☐ Behavioural & social sciences ☐ Ecological, evolutionary & environmental sciences

For a reference copy of the document with all sections, see nature.com/documents/nr-reporting-summary-flat.pdf

Life sciences study design

All studies must disclose on these points even when the disclosure is negative.

Sample size	For qPCR, immunoblotting, mRNA-sequencing, fluorescence imaging, and CUT&RUN experiments, the number of worms used was determined according to our pre-tests. For lifespan analysis, the sample size was determined according to the common standards of the C. elegans aging biology field, which typically uses a population of about 100 animals for a lifespan analysis (Wilkinson, Taylor, & Dillin, 2012; https://doi.org/10.1016/B978-0-12-394620-1.00012-6).
Data exclusions	For lifespan analysis, worms that exploded, experienced internal hatching (bagged), or crawled off the plate were "censored" in the statistical analysis. This is common practice in the C. elegans aging biology field.
Replication	All experiments were repeated with at least two biological replicates with similar results. All attempts at replication were successful.
Randomization	For all experiments in which in which worms were allocated into groups (i.e. RNAi experiments), worms were selected at random to be placed into each group.
Blinding	Blinding was not used in this study to allow for inter-group observations during the study, to facilitate ease of experiments, and because blinding was not practical. Specifically, the mutants used in this study have visible phenotypes, as do the RNAi systems used (hda-1 and glp-1), making blinding impractical.

Reporting for specific materials, systems and methods

We require information from authors about some types of materials, experimental systems and methods used in many studies. Here, indicate whether each material, system or method listed is relevant to your study. If you are not sure if a list item applies to your research, read the appropriate section before selecting a response.

Materials & experimental systems

n/a	Involved in the study	
☐	☒	Antibodies
☒	☐	Eukaryotic cell lines
☒	☐	Palaeontology and archaeology
☐	☒	Animals and other organisms
☒	☐	Clinical data
☒	☐	Dual use research of concern
☒	☐	Plants

Methods

n/a	Involved in the study	
☐	☒	ChIP-seq
☒	☐	Flow cytometry
☒	☐	MRI-based neuroimaging

Antibodies

Antibodies used	Primary Antibodies - for both CUT&RUN and immunoblotting<br>HA Tag (C29F4), Cell Signaling, #3724, Lot 10, 1:100 for CUT&RUN, 1:1000 for immunoblotting<br>FLAG (DYKDDDDK) Tag (FG4R), ThermoFisher, #MA1-91878, Lot VF298990, 1:100 for CUT&RUN, 1:1000 for immunoblotting<br>Histone H3, Abcam, #ab1791, Lot GR3252153-1, 1:100 for CUT&RUN, 1:2000 for immunoblotting<br>GFP Tag (3E6), Invitrogen, #A-11120; for IP-Mass Spec<br><br>Secondary Antibodies<br>Rabbit anti mouse IgG, Abcam, #ab46540, Lot GR3386453-2 - used for CUT&RUN, 1:100 for CUT&RUN<br>Goat anti rabbit IgG, Li-cor, #926-32211, Lot C60321-05 - used for immunoblotting, 1:5000 for immunoblotting<br>Goat anti mouse IgG, Li-cor, #926-68070, Lot C60107-03 - used for immunoblotting, 1:5000 for immunoblotting
Validation	Each antibody used in this study has been validated by the manufacturer and published elsewhere in the literature. Primary antibodies used were:

HA Tag (C29F4) - Cell Signaling #3724 - Noted on the Cell Signaling website to be used in both western blotting and CUT&RUN.The website notes that as this antibody targets the HA-tag, an exogenously expressed protein, it is expected to be reactive in all species, and it has been previously used successfully in C. elegans, including Ahier et al., 2018 - https://doi.org/10.1038/s41556-017-0023-x and Xie et al., 2022 - https://doi.org/10.1242/jcs.259396).

FLAG (DYKDDDDK) Tag (FG4R) - ThermoFisher #MA1-91878 - Noted on ThermoFisher website to be used in both western blotting and ChIP. Again, ThermoFisher's website indicates that this antibody reacts to an exogenous tag and is not species-specific. This antibody has also been used previously in C. elegans, for example in Woglar et al., 2022 - https://doi.org/10.1371/journal.pbio.3001784.

Histone H3 - Abcam #ab1791 - Noted on the Abcam website to be used in both western blotting and ChIP. This has been previously used in C. elegans in several publications, including Wang et al., 2018 - https://doi.org/10.7554/eLife.34970.

GFP Tag (3E6), Invitrogen, #A-11120 - Noted on the Invitrogen website to be used in immunoprecipitation. This antibody is also noted to react to an exogenous tag and is not species-specific. This antibody has been previously used in C. elegans for immmunoprecipitation, for example in Wei et al., 2016 - https://doi.org/10.1038/ncomms12437.

Secondary Antibodies

Rabbit anti mouse IgG, Abcam, #ab46540. This is a secondary antibody which binds to mouse IgG. It was previously been used as a negative control in C. elegans in Ikegami et al., 2010 - https://doi.org/10.1186/gb-2010-11-12-r120.

Goat anti rabbit IgG, Li-cor, #926-32211. This is a fluorescent secondary antibody, which has previously been used for immunoblotting analysis in C. elegans, as in Maduzia et al., 2011 - https://doi.org/10.1074/jbc.M110.188581.

Goat anti mouse IgG, Li-cor, #926-68070. This is a fluorescent secondary antibody, which has previously been used for immunoblotting in C. elegans, as in Xie et al., 2022 - https://doi.org/10.1242/jcs.259396.

Animals and other research organisms

Policy information about studies involving animals; ARRIVE guidelines recommended for reporting animal research, and Sex and Gender in Research

Laboratory animals	C. elegans laboratory strains in the N2 background were used. All animals were hermaphrodites. All strains are listed in Supplementary Data 5. The age of animals for each experiment is specified within the text. All CUT&RUN experiments were conducted with day 1 adults. RNA-seq experiments were conducted on day 1, day 3, and day 12 adults. IP-Mass Spec experiments were conducted with mixed stage worms. Lifespan experiments were started at day 1 of adulthood and monitored until death. Immunoblotting experiments were conducted either at day 1 of adulthood (Supplementary Fig. 3d) or at day 3 of adulthood (Supplementary Fig. 7d). Fluorescence imaging studies were conducted at either day 3 of adulthood (Supplementary Fig. 7f-i) or at day 1 of adulthood (Supplementary Fig. 8c-f).
Wild animals	Wild animals were not used.
Reporting on sex	Only hermaphrodites were used in this study.
Field-collected samples	Field-collected samples were not used.
Ethics oversight	No ethics committee was required to approve this study as it was conducted in C. elegans.

Note that full information on the approval of the study protocol must also be provided in the manuscript.

Plants

Seed stocks	Plants were not used.
Novel plant genotypes	Plants were not used.
Authentication	Plants were not used.

ChIP-seq

Data deposition

☒ Confirm that both raw and final processed data have been deposited in a public database such as GEO.

☒ Confirm that you have deposited or provided access to graph files (e.g. BED files) for the called peaks.

Data access links<br>*May remain private before publication.*	NCBI GEO database under accession code GSE224076 (CUT&RUN) under SuperSeries GSE224078 (reviewer token ypcjawiehvalxav).
Files in database submission	glp-1_FLAGantibody_rep1_R1.fastq.gz<br>glp-1_FLAGantibody_rep1_R2.fastq.gz

glp-1_FLAGantibody_rep2_R1.fastq.gz
glp-1_FLAGantibody_rep2_R2.fastq.gz
glp-1_HAantibody_rep1_R1.fastq.gz
glp-1_HAantibody_rep1_R2.fastq.gz
glp-1_HAantibody_rep2_R1.fastq.gz
glp-1_HAantibody_rep2_R2.fastq.gz
hcf-1HDA-1GFP_glp-1RNAi_H3antibody_rep1_R1.fastq.gz
hcf-1HDA-1GFP_glp-1RNAi_H3antibody_rep1_R2.fastq.gz
hcf-1HDA-1GFP_glp-1RNAi_H3antibody_rep2_R1.fastq.gz
hcf-1HDA-1GFP_glp-1RNAi_H3antibody_rep2_R2.fastq.gz
hcf-1HDA-1GFP_glp-1RNAi_HAantibody_rep1_R1.fastq.gz
hcf-1HDA-1GFP_glp-1RNAi_HAantibody_rep1_R2.fastq.gz
hcf-1HDA-1GFP_glp-1RNAi_HAantibody_rep2_R1.fastq.gz
hcf-1HDA-1GFP_glp-1RNAi_HAantibody_rep2_R2.fastq.gz
hcf-1HDA-1GFP_L4440_H3antibody_rep1_R1.fastq.gz
hcf-1HDA-1GFP_L4440_H3antibody_rep1_R2.fastq.gz
hcf-1HDA-1GFP_L4440_H3antibody_rep2_R1.fastq.gz
hcf-1HDA-1GFP_L4440_H3antibody_rep2_R2.fastq.gz
hcf-1HDA-1GFP_L4440_HAantibody_rep1_R1.fastq.gz
hcf-1HDA-1GFP_L4440_HAantibody_rep1_R2.fastq.gz
hcf-1HDA-1GFP_L4440_HAantibody_rep2_R1.fastq.gz
hcf-1HDA-1GFP_L4440_HAantibody_rep2_R2.fastq.gz
hcf-1SET-26HA_glp-1RNAi_H3antibody_rep1_R1.fastq.gz
hcf-1SET-26HA_glp-1RNAi_H3antibody_rep1_R2.fastq.gz
hcf-1SET-26HA_glp-1RNAi_H3antibody_rep2_R1.fastq.gz
hcf-1SET-26HA_glp-1RNAi_H3antibody_rep2_R2.fastq.gz
hcf-1SET-26HA_glp-1RNAi_HAantibody_rep1_R1.fastq.gz
hcf-1SET-26HA_glp-1RNAi_HAantibody_rep1_R2.fastq.gz
hcf-1SET-26HA_glp-1RNAi_HAantibody_rep2_R1.fastq.gz
hcf-1SET-26HA_glp-1RNAi_HAantibody_rep2_R2.fastq.gz
hcf-1SET-26HA_L4440_H3antibody_rep1_R1.fastq.gz
hcf-1SET-26HA_L4440_H3antibody_rep1_R2.fastq.gz
hcf-1SET-26HA_L4440_H3antibody_rep2_R1.fastq.gz
hcf-1SET-26HA_L4440_H3antibody_rep2_R2.fastq.gz
hcf-1SET-26HA_L4440_HAantibody_rep1_R1.fastq.gz
hcf-1SET-26HA_L4440_HAantibody_rep1_R2.fastq.gz
hcf-1SET-26HA_L4440_HAantibody_rep2_R1.fastq.gz
hcf-1SET-26HA_L4440_HAantibody_rep2_R2.fastq.gz
HCF-1GFP_FLAGantibody_rep1_R1.fastq.gz
HCF-1GFP_FLAGantibody_rep1_R2.fastq.gz
HCF-1GFP_FLAGantibody_rep2_R1.fastq.gz
HCF-1GFP_FLAGantibody_rep2_R2.fastq.gz
HCF-1GFP_glp1RNAi_FLAGab_rep2_R1.fastq.gz
HCF-1GFP_glp1RNAi_FLAGab_rep2_R2.fastq.gz
HCF-1GFP_glp1RNAi_H3antibody_rep2_R1.fastq.gz
HCF-1GFP_glp1RNAi_H3antibody_rep2_R2.fastq.gz
HCF-1GFP_glp-1RNAi_FLAGantibody_rep1_R1.fastq.gz
HCF-1GFP_glp-1RNAi_FLAGantibody_rep1_R2.fastq.gz
HCF-1GFP_glp-1RNAi_H3antibody_rep1_R1.fastq.gz
HCF-1GFP_glp-1RNAi_H3antibody_rep1_R2.fastq.gz
HCF-1GFPglp-1_FLAGantibody_rep1_R1.fastq.gz
HCF-1GFPglp-1_FLAGantibody_rep1_R2.fastq.gz
HCF-1GFPglp-1_FLAGantibody_rep2_R1.fastq.gz
HCF-1GFPglp-1_FLAGantibody_rep2_R2.fastq.gz
HCF-1GFPglp-1_H3antibody_rep1_R1.fastq.gz
HCF-1GFPglp-1_H3antibody_rep1_R2.fastq.gz
HCF-1GFPglp-1_H3antibody_rep2_R1.fastq.gz
HCF-1GFPglp-1_H3antibody_rep2_R2.fastq.gz
HCF-1GFP_L4440_FLAGantibody_rep1_R1.fastq.gz
HCF-1GFP_L4440_FLAGantibody_rep1_R2.fastq.gz
HCF-1GFP_L4440_FLAGantibody_rep2_R1.fastq.gz
HCF-1GFP_L4440_FLAGantibody_rep2_R2.fastq.gz
HCF-1GFP_L4440_H3antibody_rep1_R1.fastq.gz
HCF-1GFP_L4440_H3antibody_rep1_R2.fastq.gz
HCF-1GFP_L4440_H3antibody_rep2_R1.fastq.gz
HCF-1GFP_L4440_H3antibody_rep2_R2.fastq.gz
HDA-1GFP_glp-1RNAi_H3antibody_rep1_R1.fastq.gz
HDA-1GFP_glp-1RNAi_H3antibody_rep1_R2.fastq.gz
HDA-1GFP_glp-1RNAi_H3antibody_rep2_R1.fastq.gz
HDA-1GFP_glp-1RNAi_H3antibody_rep2_R2.fastq.gz
HDA-1GFP_glp-1RNAi_HAantibody_rep1_R1.fastq.gz
HDA-1GFP_glp-1RNAi_HAantibody_rep1_R2.fastq.gz
HDA-1GFP_glp-1RNAi_HAantibody_rep2_R1.fastq.gz
HDA-1GFP_glp-1RNAi_HAantibody_rep2_R2.fastq.gz
HDA-1GFPglp-1_HAantibody_rep1_R1.fastq.gz
HDA-1GFPglp-1_HAantibody_rep1_R2.fastq.gz

HDA-1GFPglp-1_HAantibody_rep2_R1.fastq.gz
HDA-1GFPglp-1_HAantibody_rep2_R2.fastq.gz
HDA-1GFP_HAantibody_rep1_R1.fastq.gz
HDA-1GFP_HAantibody_rep1_R2.fastq.gz
HDA-1GFP_HAantibody_rep2_R1.fastq.gz
HDA-1GFP_HAantibody_rep2_R2.fastq.gz
HDA-1GFP_L4440_H3antibody_rep1_R1.fastq.gz
HDA-1GFP_L4440_H3antibody_rep1_R2.fastq.gz
HDA-1GFP_L4440_H3antibody_rep2_R1.fastq.gz
HDA-1GFP_L4440_H3antibody_rep2_R2.fastq.gz
HDA-1GFP_L4440_HAantibody_rep1_R1.fastq.gz
HDA-1GFP_L4440_HAantibody_rep1_R2.fastq.gz
HDA-1GFP_L4440_HAantibody_rep2_R1.fastq.gz
HDA-1GFP_L4440_HAantibody_rep2_R2.fastq.gz
N2_FLAGantibody_rep1_R1.fastq.gz
N2_FLAGantibody_rep1_R2.fastq.gz
N2_FLAGantibody_rep2_R1.fastq.gz
N2_FLAGantibody_rep2_R2.fastq.gz
N2_HAantibody_rep1forHDA1_R1.fastq.gz
N2_HAantibody_rep1forHDA1_R2.fastq.gz
N2_HAantibody_rep1forSET26_R1.fastq.gz
N2_HAantibody_rep1forSET26_R2.fastq.gz
N2_HAantibody_rep2_R1.fastq.gz
N2_HAantibody_rep2_R2.fastq.gz
set-26HCF1GFP_glp-1RNAi_FLAGantibody_rep1_R1.fastq.gz
set-26HCF-1GFP_glp1RNAi_FLAGantibody_rep2_R1.fastq.gz
set-26HCF-1GFP_glp1RNAi_FLAGantibody_rep2_R2.fastq.gz
set-26HCF-1GFP_glp1RNAi_H3antibody_rep2_R1.fastq.gz
set-26HCF-1GFP_glp1RNAi_H3antibody_rep2_R2.fastq.gz
set-26HCF-1GFP_glp-1RNAi_FLAGantibody_rep1_R2.fastq.gz
set-26HCF-1GFP_glp-1RNAi_H3antibody_rep1_R1.fastq.gz
set-26HCF-1GFP_glp-1RNAi_H3antibody_rep1_R2.fastq.gz
set-26HCF-1GFPglp-1_FLAGantibody_rep1_R1.fastq.gz
set-26HCF-1GFPglp-1_FLAGantibody_rep1_R2.fastq.gz
set-26HCF-1GFPglp-1_FLAGantibody_rep2_R1.fastq.gz
set-26HCF-1GFPglp-1_FLAGantibody_rep2_R2.fastq.gz
set-26HCF-1GFPglp-1_H3antibody_rep1_R1.fastq.gz
set-26HCF-1GFPglp-1_H3antibody_rep1_R2.fastq.gz
/set-26HCF-1GFPglp-1_H3antibody_rep2_R1.fastq.gz
set-26HCF-1GFPglp-1_H3antibody_rep2_R2.fastq.gz
set-26HCF-1GFP_L4440_FLAGantibody_rep1_R1.fastq.gz
set-26HCF-1GFP_L4440_FLAGantibody_rep1_R2.fastq.gz
set-26HCF-1GFP_L4440_FLAGantibody_rep2_R1.fastq.gz
set-26HCF-1GFP_L4440_FLAGantibody_rep2_R2.fastq.gz
set-26HCF-1GFP_L4440_H3antibody_rep1_R1.fastq.gz
set-26HCF-1GFP_L4440_H3antibody_rep1_R2.fastq.gz
set-26HCF-1GFP_L4440_H3antibody_rep2_R1.fastq.gz
set-26HCF-1GFP_L4440_H3antibody_rep2_R2.fastq.gz
set-26HDA-1GFP_glp-1RNAi_H3antibody_rep1_R1.fastq.gz
set-26HDA-1GFP_glp-1RNAi_H3antibody_rep1_R2.fastq.gz
set-26HDA-1GFP_glp-1RNAi_H3antibody_rep2_R1.fastq.gz
set-26HDA-1GFP_glp-1RNAi_H3antibody_rep2_R2.fastq.gz
set-26HDA-1GFP_glp-1RNAi_HAantibody_rep1_R1.fastq.gz
set-26HDA-1GFP_glp-1RNAi_HAantibody_rep1_R2.fastq.gz
set-26HDA-1GFP_glp-1RNAi_HAantibody_rep2_R1.fastq.gz
set-26HDA-1GFP_glp-1RNAi_HAantibody_rep2_R2.fastq.gz
set-26HDA-1GFP_L4440_H3antibody_rep1_R1.fastq.gz
set-26HDA-1GFP_L4440_H3antibody_rep1_R2.fastq.gz
set-26HDA-1GFP_L4440_H3antibody_rep2_R1.fastq.gz
set-26HDA-1GFP_L4440_H3antibody_rep2_R2.fastq.gz
set-26HDA-1GFP_L4440_HAantibody_rep1_R1.fastq.gz
set-26HDA-1GFP_L4440_HAantibody_rep1_R2.fastq.gz
set-26HDA-1GFP_L4440_HAantibody_rep2_R1.fastq.gz
set-26HDA-1GFP_L4440_HAantibody_rep2_R2.fastq.gz
SET-26HA_glp-1RNAi_H3antibody_rep1_R1.fastq.gz
SET-26HA_glp-1RNAi_H3antibody_rep1_R2.fastq.gz
SET-26HA_glp-1RNAi_H3antibody_rep2_R1.fastq.gz
SET-26HA_glp-1RNAi_H3antibody_rep2_R2.fastq.gz
SET-26HA_glp-1RNAi_HAantibody_rep1_R1.fastq.gz
SET-26HA_glp-1RNAi_HAantibody_rep1_R2.fastq.gz
SET-26HA_glp-1RNAi_HAantibody_rep2_R1.fastq.gz
SET-26HA_glp-1RNAi_HAantibody_rep2_R2.fastq.gz
SET-26HAglp-1_HAantibody_rep1_R1.fastq.gz
SET-26HAglp-1_HAantibody_rep1_R2.fastq.gz
SET-26HAglp-1_HAantibody_rep2_R1.fastq.gz
SET-26HAglp-1_HAantibody_rep2_R2.fastq.gz

SET-26HA_HAantibody_rep1_R1.fastq.gz
SET-26HA_HAantibody_rep1_R2.fastq.gz
SET-26HA_HAantibody_rep2_R1.fastq.gz
SET-26HA_HAantibody_rep2_R2.fastq.gz
SET-26HA_L4440_H3antibody_rep1_R1.fastq.gz
SET-26HA_L4440_H3antibody_rep1_R2.fastq.gz
SET-26HA_L4440_H3antibody_rep2_R1.fastq.gz
SET-26HA_L4440_H3antibody_rep2_R2.fastq.gz
SET-26HA_L4440_HAantibody_rep1_R1.fastq.gz
SET-26HA_L4440_HAantibody_rep1_R2.fastq.gz
SET-26HA_L4440_HAantibody_rep2_R1.fastq.gz
SET-26HA_L4440_HAantibody_rep2_R2.fastq.gz
HCF-1.vs.antibodybackground_wildtype_OP50_MergedReps.bigwig
HCF-1.vs.antibodybackground_wildtype_OP50_MergedReps_narrow_peaks_peaks.narrowPeak
HCF-1.vs.antibodybackground_wildtype_OP50_MergedReps_narrow_peaks_peaks.xls
HCF-1.vs.H3_glp-1mut_OP50_MergedReps.bigwig
HCF-1.vs.H3_glp-1mut_OP50_MergedReps_narrow_peaks_peaks.narrowPeak
HCF-1.vs.H3_glp-1mut_OP50_MergedReps_narrow_peaks_peaks.xls
HCF-1.vs.H3_glp-1RNAi_MergedReps.bigwig
HCF-1.vs.H3_glp-1RNAi_MergedReps_narrow_peaks_peaks.narrowPeak
HCF-1.vs.H3_glp-1RNAi_MergedReps_narrow_peaks_peaks.xls
HCF-1.vs.H3_L4440_MergedReps.bigwig
HCF-1.vs.H3_L4440_MergedReps_narrow_peaks_peaks.narrowPeak
HCF-1.vs.H3_L4440_MergedReps_narrow_peaks_peaks.xls
HCF-1_somatic.vs.antibodybackground_glp1_OP50_MergedReps.bigwig
HCF-1_somatic.vs.antibodybackground_glp1_OP50_MergedReps_narrow_peaks_peaks.narrowPeak
HCF-1_somatic.vs.antibodybackground_glp-1_OP50_MergedReps_narrow_peaks_peaks.xls
HDA-1.vs.antibodybackground_wildtype_OP50_MergedReps.bigwig
HDA-1.vs.antibodybackground_wildtype_OP50_MergedReps_narrow_peaks_peaks.narrowPeak
HDA-1.vs.antibodybackground_wildtype_OP50_MergedReps_narrow_peaks_peaks.xls
HDA-1.vs.H3_glp-1RNAi_mergedreps.bigwig
HDA-1.vs.H3_glp-1RNAi_mergedreps_narrow_peaks_peaks.narrowPeak
HDA-1.vs.H3_glp-1RNAi_mergedreps_narrow_peaks_peaks.xls
HDA-1.vs.H3_L4440_mergedreps.bigwig
HDA-1.vs.H3_L4440_mergedreps_narrow_peaks_peaks.narrowPeak
HDA-1.vs.H3_L4440_mergedreps_narrow_peaks_peaks.xls
HDA-1_somatic.vs.antibodybackground_glp-1_OP50_MergedReps.bigwig
HDA-1_somatic.vs.antibodybackground_glp-1_OP50_MergedReps_narrow_peaks_peaks.narrowPeak
HDA-1_somatic.vs.antibodybackground_glp-1_OP50_MergedReps_narrow_peaks_peaks.xls
set-26mutant_HCF-1.vs.H3_glp-1mut_OP50_MergedReps.bigwig
set-26mutant_HCF-1.vs.H3_glp-1mut_OP50_MergedReps_narrow_peaks_peaks.narrowPeak
set-26mutant_HCF-1.vs.H3_glp-1mut_OP50_MergedReps_narrow_peaks_peaks.xls
set-26mutant_HCF-1.vs.H3_glp-1RNAi_MergedReps.bigwig
set-26mutant_HCF-1.vs.H3_glp-1RNAi_MergedReps_narrow_peaks_peaks.narrowPeak
set-26mutant_HCF-1.vs.H3_glp-1RNAi_MergedReps_narrow_peaks_peaks.xls
set-26mutant_HCF-1.vs.H3_L4440_MergedReps.bigwig
set-26mutant_HCF-1.vs.H3_L4440_MergedReps_narrow_peaks_peaks.narrowPeak
set-26mutant_HCF-1.vs.H3_L4440_MergedReps_narrow_peaks_peaks.xls
set-26mutant_HDA-1.vs.H3_glp-1RNAi_mergedreps.bigwig
set-26mutant_HDA-1.vs.H3_glp-1RNAi_mergedreps_narrow_peaks_peaks.narrowPeak
set-26mutant_HDA-1.vs.H3_glp-1RNAi_mergedreps_narrow_peaks_peaks.xls
set-26mutant_HDA-1.vs.H3_L4440_mergedreps.bigwig
set-26mutant_HDA-1.vs.H3_L4440_mergedreps_narrow_peaks_peaks.narrowPeak
set-26mutant_HDA-1.vs.H3_L4440_mergedreps_narrow_peaks_peaks.xls
SET-26.vs.antibodybackground_wildtype_OP50_MergedReps.bigwig
SET-26.vs.antibodybackground_wildtype_OP50_MergedReps_narrow_peaks_peaks.narrowPeak
SET-26.vs.antibodybackground_wildtype_OP50_MergedReps_narrow_peaks_peaks.xls
SET-26.vs.H3_glp-1RNAi_MergedReps.bigwig
SET-26.vs.H3_glp-1RNAi_MergedReps_narrow_peaks_peaks.narrowPeak
SET-26.vs.H3_glp-1RNAi_MergedReps_narrow_peaks_peaks.xls
SET-26.vs.H3_L4440_MergedReps.bigwig
SET-26.vs.H3_L4440_MergedReps_narrow_peaks_peaks.narrowPeak
SET-26.vs.H3_L4440_MergedReps_narrow_peaks_peaks.xls
SET-26_somatic.vs.antibodybackground_glp-1_OP50_MergedReps.bigwig
SET-26_somatic.vs.antibodybackground_glp-1_OP50_MergedReps_narrow_peaks_peaks.narrowPeak
SET-26_somatic.vs.antibodybackground_glp-1_OP50_MergedReps_narrow_peaks_peaks.xls
hcf-1mutant_HDA-1.vs.H3_glp-1RNAi_mergedreps.bigwig
hcf-1mutant_HDA-1.vs.H3_glp-1RNAi_mergedreps_narrow_peaks_peaks.narrowPeak
hcf-1mutant_HDA-1.vs.H3_glp-1RNAi_mergedreps_narrow_peaks_peaks.xls
hcf-1mutant_HDA-1.vs.H3_L4440_mergedreps.bigwig
hcf-1mutant_HDA-1.vs.H3_L4440_mergedreps_narrow_peaks_peaks.narrowPeak
hcf-1mutant_HDA-1.vs.H3_L4440_mergedreps_narrow_peaks_peaks.xls
hcf-1mutant_SET-26.vs.H3_glp-1RNAi_MergedReps.bigwig
hcf-1mutant_SET-26.vs.H3_glp-1RNAi_MergedReps_narrow_peaks_peaks.narrowPeak
hcf-1mutant_SET-26.vs.H3_glp-1RNAi_MergedReps_narrow_peaks_peaks.xls
hcf-1mutant_SET-26.vs.H3_L4440_MergedReps.bigwig

hcf-1mutant_SET-26.vs.H3_L4440_MergedReps_narrow_peaks_peaks.narrowPeak
hcf-1mutant_SET-26.vs.H3_L4440_MergedReps_narrow_peaks_peaks.xls

Genome browser session (e.g. UCSC)	*Provide a link to an anonymized genome browser session for "Initial submission" and "Revised version" documents only, to enable peer review. Write "no longer applicable" for "Final submission" documents.*

Methodology

Replicates	Two biological replicates were obtained for all CUT&RUN experiments, and the correlation analysis for each experiment is provided as figures within the manuscript.
Sequencing depth	>5 million reads were obtained for each CUT&RUN experiment (as has been shown to be appropriate for CUT&RUN (https://doi.org/10.7554/eLife.21856)). Sequencing reads were 2x32 paired-end sequencing with an Illumina NextSeq 500 machine. Reads were typically less around 50% or less duplicated, however reads appearing as duplicates are believed to be in part due to the precise nature of MNase cleavage in CUT&RUn experiments (https://doi.org/10.1186/S12864-018-4933-1 and https://doi.org/10.1186/S13059-019-1802-4).
Antibodies	Antibodies are provided in Supplementary Data 5.
Peak calling parameters	Indexed bam files were used for narrow peak calling with MACS2 (v2.1.4) using the settings -f BAM - g ce –call-summits –keep-dup all -q 0.01 -m 5 50 –nomodel. The control files used (either antibody background or H3 CUT&RUN) are detailed in the Supplementary Methods section of the Supplementary Information File.
Data quality	Adaptor sequences were trimmed and low quality reads were filtered out from sequencing files using Trim Galore! (v0.6.5), which utilizes Cutadapt (v3.4) and FastQC (v0.11.8), with the settings –paired –q 20 –fastqc. Only properly paired and aligned sequences were kept after the alignment process. A q value of 0.01 was used in peak calling parameters in MACS2. Correlation analyses for all CUT&RUN replicates are shown within supplementary figures of the manuscript. Bigwig files were also manually inspected for all experiments in IGV and showed similar results in each experiment.
Software	All software used for data analysis including version number is included within the manuscript and in the Supplementary Methods section of the Supplementary Information File.
